# Selective Binding and Elution of Aptamers for Pesticides Based on Sol-Gel-Coated Nanoporous Anodized Aluminum Oxide Membrane

**DOI:** 10.3390/nano10081533

**Published:** 2020-08-05

**Authors:** Eun Seob Lim, Min-Cheol Lim, Kisang Park, Gaeul Lee, Jeong-A Lim, Min-Ah Woo, Nari Lee, Sung-Wook Choi, Hyun-Joo Chang

**Affiliations:** 1 Research Group of Consumer Safety, Research Division of Strategic Food Technology, Korea Food Research Institute, Wanju-gun, Jeollabuk-do 55365, Korea; 50004@kfri.re.kr (E.S.L.); mclim@kfri.re.kr (M.-C.L.); 07938@kfri.re.kr (K.P.); 50045@kfri.re.kr (G.L.); jeongalim@kfri.re.kr (J.-A.L.); mawoo@kfri.re.kr (M.-A.W.); nari@kfri.re.kr (N.L.); swchoi@kfri.re.kr (S.-W.C.); 2 Department of Food Biotechnology, Korea University of Science and Technology, Daejeon 34113, Korea; 3 Department of Molecular Science and Technology, Ajou University, Suwon-si, Gyeonggi-do 16499, Korea; 4 Department of Food Science and Technology, Chonbuk National University, Jeonju-si, Jeollabuk-do 54896, Korea

**Keywords:** anodized aluminum oxide, sol-gel, aptamer, SELEX, pesticides

## Abstract

Sol-gel-based mesopores allow the entry of target small molecules retained in their cavity and aptamers to bind to target molecules. Herein, sol-gel-based materials are applied to screen-selective aptamers for small molecules, such as pesticides. To enhance the efficiency of aptamer screening using a sol-gel, it is necessary to increase the binding surface. In this study, we applied the sol-gel to an anodized aluminum oxide (AAO) membrane, and the morphological features were observed via electron microscopy after spin coating. The binding and elution processes were conducted and confirmed by fluorescence microscopy and polymerase chain reaction. The sol-gel coating on the AAO membrane formed a hollow nanocolumn structure. A diazinon-binding aptamer was bound to the diazinon-containing sol-gel-coated AAO membrane, and the bound aptamer was effectively retrieved from the sol-gel matrix by thermal elution. As a proof of concept, a sol-gel-coated AAO disc was mounted on the edge of a pipette tip, and the feasibility of the prepared platform for the systematic evolution of ligands by exponential enrichment (SELEX) of the aptamer binding was also confirmed. The proposed approach will be applied to an automated SELEX cycle using an automated dispenser, such as a pipetting robot, in the near future.

## 1. Introduction

In modern agriculture, pesticides are widely and extensively used to protect crops and farm animals against insects and pests [1]. Despite the various beneficial effects of agrochemical use, such as high production yield and food safety, environmental issues are vastly increasing in several developed and developing countries [2,3]. The overuse of pesticides causes critical problems in public health and the environment through the contamination of water, soil, and agricultural products. Consequently, strict mandatory regulations have been implemented to regulate inappropriate disposals of pesticides and their residues. For example, the Positive List System (PLS) was introduced as a mandatory regulation for the safety management of residual pesticides in agricultural products in Korea in 2019 [4]. Under the supervision of the PLS, all pesticides are uniformly limited to below 0.01 mg/kg, except for cases wherein maximum residue limits (MRLs) are established. Although the management system has strengthened the enforcement of residue limits, the development of effective and rapid technologies for detecting pesticides in food and environmental samples remains essential.

In recent years, several studies have reported systematic methods based on conventional analytical instruments and novel biosensors. These traditional analytical methods, recognized as the gold standard for determining pesticide residues in food samples, are very effective and fairly accurate; they comprise high-performance liquid chromatography (HPLC) [5,6], gas chromatography [7,8], mass chromatography [9,10], and their combinations [11,12]. Despite the advantages of conventional analytical methods, they are insufficient to be efficiently applied for on-site and rapid detection because of limitations such as time and labor consumption, complex procedures, and expensive equipment. However, broad pesticide-targeted enzyme reaction-based colorimetric biosensors have been developed for simple and on-site detection [13,14]. Nonetheless, these enzyme-based detection approaches have limitations in the discrimination of individual pesticides in food samples. Therefore, the development of biosensors using target pesticide-specific ligands is necessary under the strengthened supervision system for pesticide residues.

Aptamers are single-stranded oligonucleotides (DNA or RNA) that form specific three-dimensional (3D) structures depending on the nucleotide sequence and specifically bind to various organic and inorganic compounds, proteins, and bacteria [15,16,17,18]. Aptamers can bind to target molecules with high specificity and affinity according to their 3D structures. Thus, they have attracted considerable attention for their high stability; wide range of targets, especially small molecules; and easy synthesis and amplification compared with protein-based antibodies [19,20]. From this perspective, aptamers are recognized as ideal alternatives for developing biosensors, referred to as aptasensors, for the detection of pesticide residues [21,22,23]. Target-specific aptamers are selected from an artificially constructed random oligonucleotide library by a method known as the systematic evolution of ligands by exponential enrichment (SELEX) [24,25]. Previously, SELEX methods for small target molecules were mainly accompanied by chemical changes using functional groups [26,27,28]. However, because this method does not completely expose the target material, there is a limit of the affinity toward selected aptamers.

Herein, we propose that sol-gel and nanoporous membrane-based SELEX platforms can be used to select aptamers against target pesticides. In this study, we fabricated a sol-gel-based nanocolumn structure for the selection of specific aptamers against pesticides. To prepare the sol-gel nanocolumn, an anodized aluminum oxide (AAO) membrane was selected as the nanoporous template. The sol-gel structure was confined to the inner surface of the AAO membrane by a simple spin-coating process. The sol-gel-based nanocolumn structure was applied to specific oligonucleotide binding and elution for entrapped pesticides in the sol-gel matrix. The structure of the fabricated sol-gel nanocolumn was investigated by scanning electron microscopy (SEM) and transmission electron microscopy (TEM). Thus, the selective binding and elution of the aptamer were evaluated using diazinon as a model pesticide and aptamer as the target pesticide. This approach will be used as a pipette-tip-mounted SELEX platform for automated liquid handling systems in the near future.

## 2. Materials and Methods 

### 2.1. Sol-Gel Coating on Nanoporous AAO Membrane

**Figure 1:**
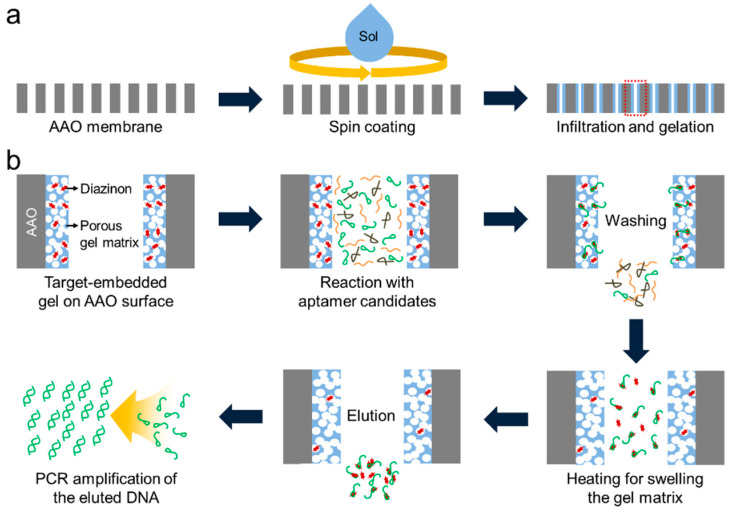
(**a**) Schematic of the procedure for performing the sol-gel coating process on the anodized aluminum oxide (AAO) membrane by spin coating and (**b**) sol-gel nanocolumn-based target pesticide-specific aptamer binding, elution, and polymerase chain reaction (PCR) amplification.

The overall sol-gel coating process on the nanoporous AAO template is shown in Figure 1a. The sol-gel composition and preparation method followed the manufacturer’s manual with the SolB complete kit (PCL Inc., Seoul, Korea). The sol-gel mixture was prepared with a composition of SolB1 (22 μL), SolB2 (8 μL), SolBH (10 μL), SolBS (10 μL), and distilled water 20 (μL); briefly spun down; and incubated in a −20 °C freezer for 30 min. To entrap the target diazinon as a model pesticide within the sol-gel matrix, diazinon (AccuStand, New Haven, CT, USA) was diluted to 100 ppm in 50% dimethylformamide, and the prepared sol-gel mixture was mixed at a volume ratio of 1:7 and briefly spun down. After fixing the AAO membrane with a 0.2-µm pore diameter (Anodisc, 25-mm diameter, 60-µm thickness, Whatman, Maidstone, UK) to a spin coater (ACE-200, Dong Ah Tech, Seoul, Korea), 80 μL of the diazinon-containing sol-gel mixture was deposited via a single-step spin coating process at 3000 rpm for 30 s onto the AAO membrane. After post-gelation overnight at high humidity (90% RH) and room temperature, the sol-gel-coated AAO membrane was cut with a 6-mm punch (Acu-punch; Acuderm, Fort Lauderdale, FL, USA) for further use. A piece of AAO membrane with a diameter of 6 mm was used as the aptamer selection substrate in the binding and elution experiments.

### 2.2. Characterization of Sol-Gel-Coated AAO Membranes

The morphology of the prepared sol-gel-coated AAO membrane was investigated by SEM (JSM-6710F, JEOL, Tokyo, Japan). After post-gelation, the samples were dried overnight in a vacuum chamber to eliminate water from the sol-gel matrix and thereafter transferred to the carbon tape on the SEM specimen stub. Subsequently, the prepared samples were coated with platinum to reduce the charging effects during SEM analysis. The structure of sol-gel nanocolumn defined by the AAO membrane was also observed by TEM (H-7650, Hitachi, Japan) after dissolving the AAO template using a 1 M NaOH solution for 3 h at 30 °C. The white precipitate upon completing the AAO dissolution reaction in the sample tube was collected by mild centrifugation with a washing step using filtered distilled water. Finally, the acquired sol-gel nanocolumn was dispersed by mild pipetting and drop-casted onto a carbon 200 mesh copper grid (Ted Pella, Redding, CA, USA). The prepared samples were dried overnight in a vacuum desiccator and observed by TEM under optimized conditions.

### 2.3. Pesticide-Specific Aptamer Binding Assay

For the aptamer binding assay, the diazinon-specific aptamer labeled with Texas Red was obtained by chemical synthesis and HPLC-purified by Bioneer Corporation (Daejeon, Korea). The anti-diazinon aptamer was a 72-mer oligonucleotide with the following sequence: 5′-ATC CGT CAC ACC TGC TCT AAT ATA GAG GTA TTG CTC TTG GAC AAG GTA CAG GGA TGG TGT TGG CTC CCG TAT-Texas Red-3′, as described in a previous report [29]. The aptamer solution was prepared in 1 × SELEX buffer (50 mM Tris-HCl, 150 mM NaCl, 25 mM KCl, 5 mM MgCl_2_, 1 mM CaCl_2_, 0.01% Tween-20, and pH 7.5) and used at a final concentration of 1 μM. The prepared sol-gel-coated AAO membrane pieces with a diameter of 6 mm were treated with 20 μg/mL yeast tRNA (Applied Biosystems, Foster City, CA, USA) in 1 × PBS (phosphate-buffered saline) (pH 7.4) at room temperature for 2 h to block the surface of the sol-gel matrix and washed in the order of 1 × PBS containing 0.2% Tween-20 and 1 × PBS buffer solution. The prepared diazinon-specific aptamer solution was heated at 95 °C for 10 min, cooled at room temperature for 2–3 h, treated on the tRNA-based blocked sol-gel-coated AAO substrates for 1 h at room temperature, and then washed three times with 1 × PBS containing 0.2% Tween-20 and 1 × PBS buffer solution. Subsequently, the washed AAO membranes were dried for 1 h in the dark, and fluorescence images were obtained using Nikon Eclipse 80i w/DS-Fi1, epi-fluorescence microscopy w/DS-Fi1 camera head (Nikon, Tokyo, Japan) at Ex: 560 nm and Em: 630 nm.

### 2.4. Thermoresponsive Aptamer Elution from Sol-Gel Matrix

The sol-gel-coated AAO membranes, after the target-specific aptamer binding process, were heated in 50 μL of deionized distilled water for 5 min at 95 °C. Subsequently, the AAO membranes were washed twice using the 1 × PBS buffer solution, and the aptamer selection substrates were reanalyzed by fluorescence microscopy (Eclipse 80i, Nikon, Tokyo, Japan) at Ex: 560 nm and Em: 630 nm. The thermo-eluated aptamers were amplified by polymerase chain reaction (PCR) with forward primer (5’- ATC CGT CAC ACC TGC TCT AAT-3’), reverse primer (5’-ATA CGG GAG CCA ACA CCA T-3’), and premix Taq™ (Ex Taq Version 2.0, Takara, Otsu, Shiga, Japan). The primers were selected using the NCBI primer-blast to cover the entire aptamer (72 bp), and in the case of reverse primer, it is the primer that covers 3′ end of the aptamer used. The PCR cycle was as follows: initial heating to 94 °C for 5 min; 25 cycles of 94 °C for 30 s, 54 °C for 30 s, and 72 °C for 30 s; and final elongation at 72 °C for 10 min. The PCR amplicons were analyzed by electrophoresis on a 5% (*w*/*v*) agarose gel. 

### 2.5. AAO Disc-Mounted Pipette Tip-Based Aptamer Binding and Elution

For the practical application of the sol-gel-coated AAO membrane as a SELEX platform, the AAO membrane, after sol-gel coating with the target pesticide, was diced as a circular disc using a specimen punch with a 3-mm diameter. Subsequently, the prepared AAO disc was mounted on the edge of a 1-mL pipette tip using a glue and stored overnight in a desiccator. Pesticide-specific binding and thermoresponsive elution of the aptamer were performed as described above. All the binding solutions, such as the aptamer solution, washing buffer, and elution buffer, were infiltrated and rejected through the AAO disc-mounted pipette tip using a manual single-channel pipette. The penetration of the solution through the fabricated AAO disc-mounted pipette tip was tested using a 1% fluorescent sodium salt solution. Target-specific aptamer binding and elution were observed via fluorescence microscopy.

## 3. Results and Discussion

Sol-gel is a silicate-based compound that forms pore sizes from nanoscale to microscale through different chemical compositions and preparation reactions [30,31]. These pores allow the aptamer to enter and bind to the desired target molecules in the cavity, thereby allowing for the selection of the aptamer according to the target-specific binding reaction [32,33]. In this study, we applied a novel immobilization method to maximize the effectiveness of sol-gel structures as a SELEX platform for small target molecules. To fabricate the desired sol-gel structure in this study, a nanoporous AAO membrane was used as a template structure to immobilize the sol-gels by capillary force-driven infiltration to nanochannels and spontaneous gelation on the inner surface of confined nanochannels (Figure 1a). The prepared sol-gel-coated AAO membrane containing pesticides in the porous cavity could react with aptamer molecules, and non-specific candidates could be eliminated by washing. Subsequently, the desired aptamer molecules toward target pesticides could be collected by a thermoresponsive elution process (Figure 1b). 

**Figure 2:**
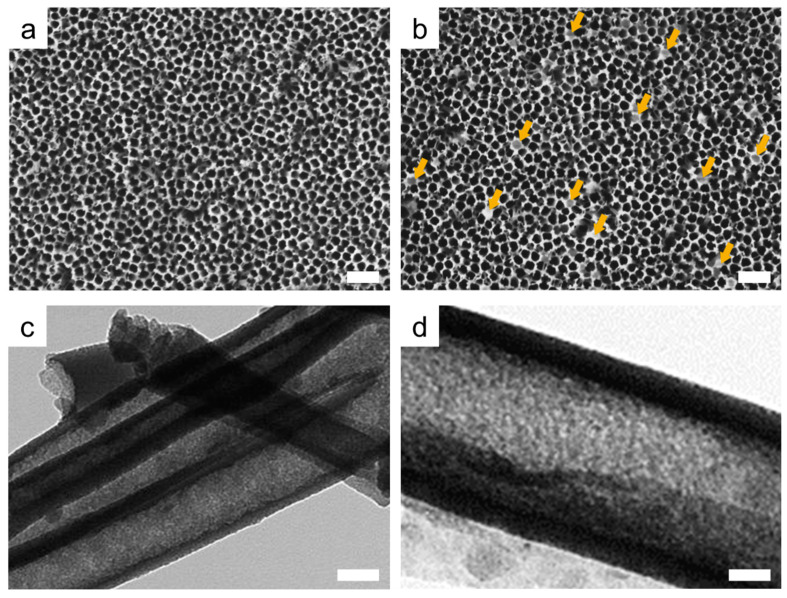
Morphological structure of pristine and sol-gel-coated anodized aluminum oxide (AAO) membranes. (**a**) Top-view scanning electron microscopy (SEM) images of pristine AAO membrane (**b**) and sol-gel-coated AAO membrane (scale bars denote 1 μm). (**c**,**d**) Constructed sol-gel nanocolumn formed on the AAO nanochannel and observed by TEM after dissolving the AAO using a NaOH buffer. Scale bars denote 100 nm and 50 nm, respectively.

The morphology of the upper surface of the AAO membrane after the sol-gel spin-coating and the gelated layer were analyzed by electron microscopy. From the SEM images of the AAO membrane, nanopores with diameters of about 200 nm were observed for the pristine template (Figure 2a). After coating the AAO membrane with sol-gel, the opening of the few nanopores was enveloped by the gelated thin film (yellow arrows), while the other nanopores maintained the nanopore opening structure (Figure 2b). This result indicated that the binding solutions, such as the aptamer solution and washing buffer, could penetrate through the nanopores of the AAO membrane, even after spin coating with the sol-gel. Upon dissolving the AAO membrane after sol-gel coating with NaOH solution, the remaining white precipitants were observed by TEM. From the TEM images, columns with a diameter of approximately 200 nm were observed, and the thickness of the gelated thin walls was approximately 20–30 nm (Figure 2c). In the high-magnification TEM image, the layer of the gelated column was composed of numerous tiny pores smaller than 5 nm (Figure 2d). Due to the thinness of sol-gel coating, the sol-gel coating AAO membrane was not clearly discriminated by SEM as shown in Figure 2a,b. Structural features of AAO membranes, such as nanometer-range pore size, high aspect ratio, and density, increase the surface area up to 1000 times to provide a sufficiently large surface area for high reactivity [34]. In addition, the gelated thin layer formed on the large surface area of the inner surface of the AAO membrane also has a mesoporous structure, thereby providing an improved target-specific aptamer selection process [35,36]. Therefore, the sol-gel-coated AAO membrane with such a large binding surface area is expected to increase the efficiency of SELEX over flat binding surfaces. 

**Figure 3:**
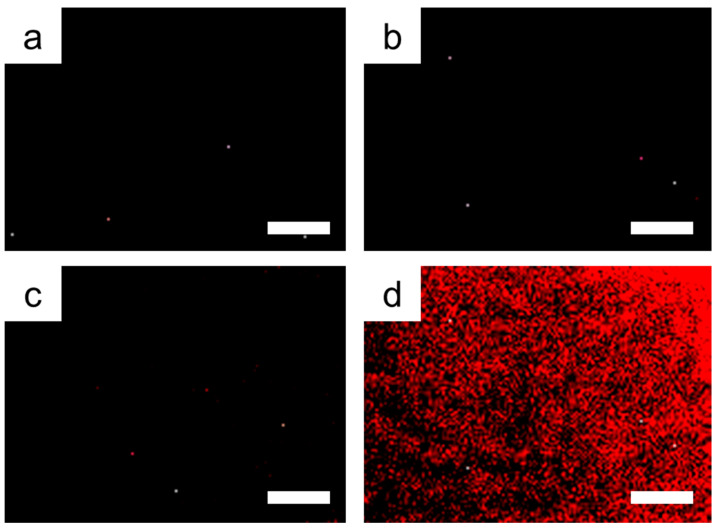
Fluorescence microscopy images of bound aptamers to diazinon-containing sol-gel-coated AAO membranes. (**a**) Binding test with pristine AAO membrane, (**b**) bare sol-gel-coated AAO membrane, (**c**) non-target atrazine containing sol-gel-coated AAO membrane, and (**d**) target diazinon containing sol-gel-coated AAO membrane. Scale bars denote 300 μm.

To confirm the feasibility of the sol-gel-coated AAO membrane for target-specific aptamer screening, diazinon, as a model pesticide, was confined in the gelated thin layer on the inner surface of AAO membrane and was tested with the fluorescence-tagged diazinon aptamer. The target diazinon-specific aptamer was reacted, and unbound DNA molecules were washed out using a washing buffer. Next, the AAO membranes were observed by fluorescence microscopy. When the target-specific aptamer was treated on the diazinon-containing sol-gel-coated AAO membrane, it exhibited a strong red fluorescence due to the tagged Texas Red dye (Figure 3d). The pristine AAO membrane and bare sol-gel-coated AAO membrane showed no fluorescence signal after treatment with the fluorescence-tagged diazinon aptamer and washed with buffer solution (Figure 3a,b). Thus, the non-target pesticide atrazine containing the sol-gel-coated AAO membrane also showed no fluorescence when treated with diazinon-specific aptamer (Figure 3c). This result indicated that the target molecule containing the sol-gel-coated AAO membrane has the ability to selectively bind small molecules in the cavity of the gelated matrix and DNA molecule. 

**Figure 4:**
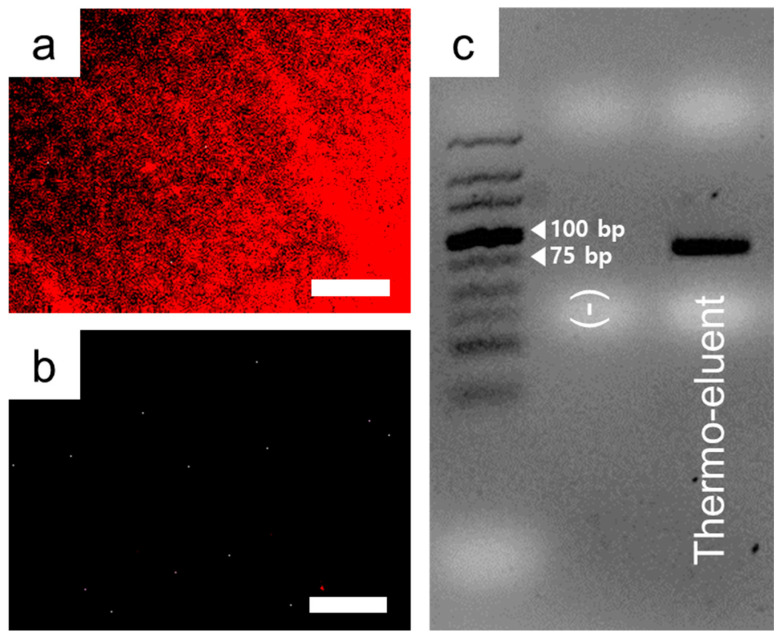
Thermo-elution test for the retrieval of bound aptamers from the target diazinon-containing sol-gel-coated AAO membrane. Fluorescence microscopy images (**a**) before and (**b**) after thermo-elution. (**c**) PCR test for thermo-eluent. Scale bars denote 300 μm.

The bound aptamer toward the target pesticide in the sol-gel-coated AAO membrane was eluted by thermal treatment, and the eluent was used as a template for PCR. From the fluorescence microscopy images, the disappearance of red fluorescence on the diazinon-containing sol-gel-coated AAO membrane after thermo-elution indicates that the target diazinon-bound aptamer effectively fell off from the mesoporous gelated thin layer (Figure 4a,b). The thermal eluent was tested as a template and amplified by PCR with the primers targeting the diazinon aptamer. The PCR product (72 bp) for the amplification of the diazinon aptamer was confirmed by agarose gel electrophoresis (Figure 4c). In previous studies, porous silicon sol-gel also successfully trapped small molecules and used them to identify target-specific aptamers that bind to xanthine [33]. The sol-gel method is advantageous for improving binding affinity by solving the exposure problem of small molecules without immobilization on solid surfaces or bigger molecules. For elution of target-specific bound aptamers from sol-gel-coated AAO membranes in this study, the thermo-elution process was successfully applied, and the feasibility of PCR-based amplification was also confirmed. 

**Figure 5:**
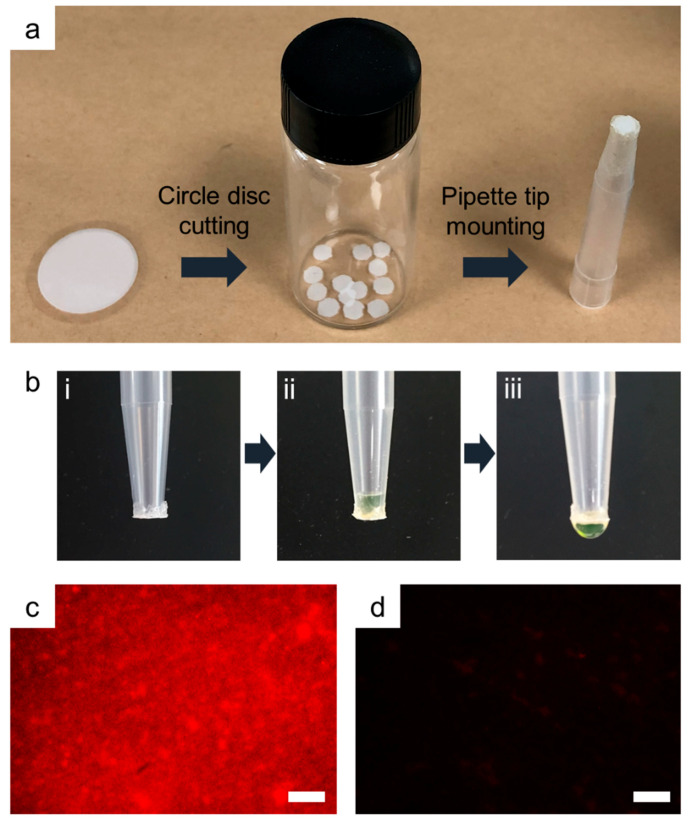
Selective binding and elution test for sol-gel-coated AAO disc-mounted pipette tip. (**a**) Preparation of sol-gel-coated AAO disc-mounted pipette tip by simple cutting and glue bonding, (**b**) solution infiltration and rejection through the prepared AAO disc-mounted pipette tip, (**c**) fluorescence microscopy image of bound aptamers to diazinon-containing sol-gel-coated AAO disc, and (**d**) that after thermal elution. Scale bars denote 500 μm.

To utilize the proposed AAO membrane-based aptamer selection as a SELEX platform, the prepared sol-gel-coated AAO membrane was mounted on a commercial pipette tip to reduce handling time and maximize the nanochannel-based binding efficiency. The sol-gel-coated AAO membrane was cut into small circular discs of the desired diameter using a sample punch and attached to the edge of the pipette tip using glue (Figure 5a). Then, the sol-gel-coated AAO disc-mounted pipette tip was tested for solution penetration across the attached disc using a manual pipette. The solution passes through the inner wall of the tubular sol-gel formed on the inner wall of the nanopore of AAO and elutes from one AAO surface to the other, while the flow rate was lower than the normal tip (Figure 5b). This result indicates that the customized tip could transport solutions by modulating the pressure of the inner space of the tip using a manual pipette. To demonstrate the sol-gel-coated AAO disc-mounted pipette tip as a SELEX platform, the target-specific binding and elution processes were performed and observed by fluorescence microscopy. Consequently, the diazinon-specific aptamer could bind to the AAO disc (Figure 5c) step-by-step as described in Figure 1 as a flow by pipetting of the solutions. Thus, the bound aptamer was also eluted by heating the solution and rejected using a pipette (Figure 5d). Several methods have been reported for separating aptamers from aptamer-target binding in SELEX cycles [37,38]. In previous studies, it was confirmed that the thermo-elution method was effectively applied to SELEX using sol-gel as the binding matrix [33]. Effective separation of the combined aptamers is important for saving resources and reducing the required process time. The heating process appears to act on proper aptamer retrieval by swelling the sol-gel matrix and dissociating the aptamer-target bonds. Furthermore, the sol-gel-coated AAO disc-mounted tip will be used for the auto-SELEX platform combined with an automated dispenser, such as a pipetting robot.

## 4. Conclusions

For effective SELEX development for small molecules, such as pesticides, the use of chemical coupling methods with chemical modifications may cause affinity loss. Thus, the utilization of sol-gel as a binding matrix guarantees enhanced interaction between the aptamer target in the cavity formed on the gelated layer. In addition, to maximize the binding efficiency, a nanoporous AAO membrane was used as a sol-gel construction platform to increase the binding surface for aptamer binding to the retained pesticide. Furthermore, the bound aptamer was confirmed to be sufficiently retrieved by simple heat treatment. Although we have not actually developed a sensor, the aptamers that have been well screened by the proposed method in this study and can be used as a target recognition material. As a proof of concept, the sol-gel-coated AAO disc-mounted pipette tip was employed as a SELEX platform using a manual pipette. This method will facilitate the automation of SELEX using existing automated pipetting equipment. This approach could be used for SELEX aptamer screening for small molecules, such as pesticides, metabolites, and toxins. Thus, the proposed platform will be applied to the automated SELEX cycle by combining it with a robotics-based sample dispenser in the near future. Automated SELEX will provide a cornerstone to provide rapid detection of continuously occurring human hazards by rapidly selecting the appropriate aptamers.
